# The Development of the Tool for Rating the qUality of Services sTandards (TRUST)

**DOI:** 10.1111/jebm.70173

**Published:** 2026-07-30

**Authors:** Zhenggang Bai, Yumeng Zhu, Xingsu Gao, Nannan Shi, Xiaoliang Cheng, Ruhai Bai, Jingchun Fan, Janne Estill, Weijie Xing, Feng Tong, Dwi Linna Suswardany, Paul Montgomery‐Marks, Liang Du, Iris Chi, Haibo Cheng, the TRUST Working Group

**Affiliations:** ^1^ School of Medicine Institute of Clinical Research Nanjing University of Chinese Medicine Nanjing China; ^2^ Campbell China Network Coordinator Center Nanjing China; ^3^ Department of Social Work School of Social Development and Public Policy Fudan University Shanghai China; ^4^ Institute of Basic Research in Clinical Medicine China Academy of Chinese Medical Sciences Beijing China; ^5^ Qlife Precision Medicine Center Northwest University Xi'an China; ^6^ School of Public Affairs Nanjing University of Science and Technology Nanjing China; ^7^ Center for Evidence‐Based Medicine Gansu University of Chinese Medicine Lanzhou China; ^8^ Gansu Province Evidence‐Based Centre for Traditional Chinese Medicine Lanzhou China; ^9^ Institute of Global Health University of Geneva Geneva Switzerland; ^10^ School of Basic Medical Sciences Lanzhou University Lanzhou China; ^11^ School of Nursing Fudan University Shanghai China; ^12^ College of International Law and Sociology Sichuan International Studies University Chongqing China; ^13^ Chronic Disease Research Center University of Muhammadiyah Surakarta Surakarta Indonesia; ^14^ Department of Social Policy, Sociology and Criminology University of Birmingham Birmingham UK; ^15^ Chinese Evidence‐Based Medicine Center West China Hospital of Sichuan University Chengdu China; ^16^ Suzanne Dworak‐Peck School of Social Work University of Southern California Los Angeles USA; ^17^ Sau Po Centre on Aging University of Hong Kong Hong Kong China; ^18^ The First Clinical Medical College Jiangsu Collaborative Innovation Center of Traditional Chinese Medicine in Prevention and Treatment of Tumor Nanjing University of Chinese Medicine Nanjing China; ^19^ Department of Oncology Affiliated Hospital of Nanjing University of Chinese Medicine Jiangsu Province Hospital of Chinese Medicine Nanjing China

**Keywords:** evidence‐based medicine, indicator, quality control, quality of healthcare

## Abstract

**Background:**

This study aimed to develop a preliminary framework for the systematic, multi‐dimensional evaluation of service standards, the Tool for Rating the qUality of Service sTandards (TRUST), to address the heterogeneity and quality‐related deficiencies in existing standards and to provide a preliminary evidence‐informed basis for evaluating, optimizing, and promoting the quality of service standards.

**Methods:**

TRUST was developed through an evidence based multi‐step process. First, a TRUST Working Group, consisting of a drafting team, an expert panel, and a steering committee, was established. A pool of candidate items was then constructed through a systematic literature review that identified 16 existing standard evaluation frameworks, followed by a group discussion. Subsequently, a structured online expert survey was conducted to assess expert agreement on the preliminary set of items. Item weights were calculated using the Precedence Chart method. Finally, we conducted a pilot study and calculated the inter‐rater reliability coefficients applying TRUST to a sample of 51 published standards.

**Results:**

TRUST comprises 17 items across five distinct dimensions: collaboration (28.0%), scope and purpose (7.6%), quality management (39.1%), implementation and updates (15.6%) and social impact (9.7%). Inter‐rater reliability (intraclass correlation coefficient [ICC] (A,1)) across items ranged from 0.502 to 0.860, and the total score showed excellent reliability (ICC (A,1) = 0.832, 95% CI: 0.723–0.901).

**Conclusion:**

TRUST offers an initial evidence‐based architecture to guide the future development of assessment instruments for standards, laying the groundwork for theoretical and practical support in optimizing and refining standards.

## 1 Introduction

Service standards are increasingly recognized by governments and industries as essential instruments for improving the quality, consistency, and accountability of services [1]. Service standards apply to a wide range of fields including but not limited to healthcare, social services, and education, exerting a defining influence on the quality and equity of both public and private services. The International Organization for Standardization (ISO) defines service standards as requirements or specifications that guarantee that services remain fit for their purpose and achieve satisfaction among customers and citizens [2]. At present, the formulation and implementation processes of service standards have already been extensively studied [3, 4, 5]. Despite the widespread recognition of the significance of service standards in the provision of high‐quality services, the systematic appraisal of the inherent quality of the standards themselves has received insufficient academic attention and practical emphasis [6, 7].

The current standard assessment work can generally be organized into four core dimensions: normative assessment, compatibility assessment, scientific assessment, and implementation effect assessment. Normative assessment focuses on the compliance of the drafting procedure and textual expression with established rules and formatting requirements [8]; compatibility assessment examines whether a standard is consistent with existing laws, regulations, and mandatory standards [9]; scientific assessment addresses whether the standard is adequately grounded in investigations, evidence, and expert consensus; and implementation effect assessment evaluates the practical value of the standard after its adoption, including its economic, social, and ecological benefits [10, 11, 12]. These dimensions provide an important analytical foundation, but they also reveal that the quality of service standards is a complex construct that extends far beyond formal compliance alone.

A variety of methods have been adopted in the assessment of standards, including direct comparison, government‐led evaluation approaches, mathematical evaluation, and scientific evaluation tools. Direct comparison methods evaluate the rationality of standards through horizontal or temporal comparisons [13]; government‐led approaches tend to rely on macro‐level policy frameworks and lack empirical supervision [14]; mathematical methods include quantitative techniques such as analytic hierarchy process, comprehensive scoring, fuzzy comprehensive evaluation, and regression analysis [15]; and scientific evaluation tools, such as AGREE [16] and STAR [17] in the field of healthcare guidelines, assess the rigorousness of the methodology [18]. Collectively, these approaches have enriched the assessment landscape, but a dedicated, integrated, and evidence‐based tool specifically designed for service standards has not yet been generated.

More importantly, current research on the assessment of service standards faces three major limitations. First, existing dimensions remain fragmented, focusing on isolated aspects rather than the full life cycle. Second, evidence‐based principles remain insufficiently emphasized, with assessments still relying heavily on expert experience, administrative judgment, or subjective scoring [19]. Third, social value and sustainability are often neglected in favor of technical compliance. Yet service standards are inherently public‐facing instruments whose quality is closely tied to whether they can serve diverse populations fairly and sustainably [20].

Without a rigorous appraisal framework, it is challenging to determine whether a service standard is merely procedurally compliant or methodologically trustworthy. Inadequate appraisal of service standard quality may impede the iterative improvement of standards, compromise the implementation of standard‐based policies [21], hinder the standardized and high‐quality delivery of services, and ultimately weaken service development for the public and society as a whole [19].

In this study, we use the term “service standards” to refer to normative documents intended to guide the design, delivery, quality assurance, evaluation, or improvement of services. This functional definition includes documents that establish recommendations, requirements, or procedural expectations for service provision. Importantly, although service standards differ across sectors, some higher‐level quality features, such as clarity of scope and purpose, interest‐holder heterogeneity, process transparency, methodological justification, implementation planning, and accountability for outcomes, may be similar across multiple service domains. These common features suggest the conceptual feasibility of a cross‐sector evaluative framework, while not implying that the specific operationalization of each item will be identical across all sectors [19]. As service systems become more complex and expectations for accountability, fairness, and effectiveness continue to rise, reliance on fragmented or experience‐based assessment approaches is no longer sufficient.

Therefore, there is an urgent need to develop a preliminary conceptual framework that may inform the future development of a multidimensional, evidence‐based, and operational instrument for appraising the intrinsic quality of service standards in a comprehensive and standardized manner. Prior research evaluating nearly 200 service standards found considerable variation in methodological rigor, limited international compatibility, and inadequate mechanisms for continuous improvement. These shortcomings have prompted calls for tools grounded in evidence‐based principles [19]. However, to the best of our knowledge, there exists currently no dedicated instrument for systematically evaluating the full life‐cycle quality of service standards that would use a common conceptual architecture relevant across multiple service fields. This study presents the initial development of the Tool for Rating the qUality of Service sTandards (TRUST), which aims to close this gap. At this stage, TRUST is intended to provide an evidence‐informed conceptual structure with cross‐sector potential, rather than to claim demonstrated applicability across all service sectors. In this sense, TRUST is intended not only to identify deficiencies in existing service standards, but also to provide methodological guidance for future development, revision, and implementation of standards. The following sections describe the preparatory work and development process of TRUST and present the tool and its items in detail.

## 2 Methods

**FIGURE 1:**
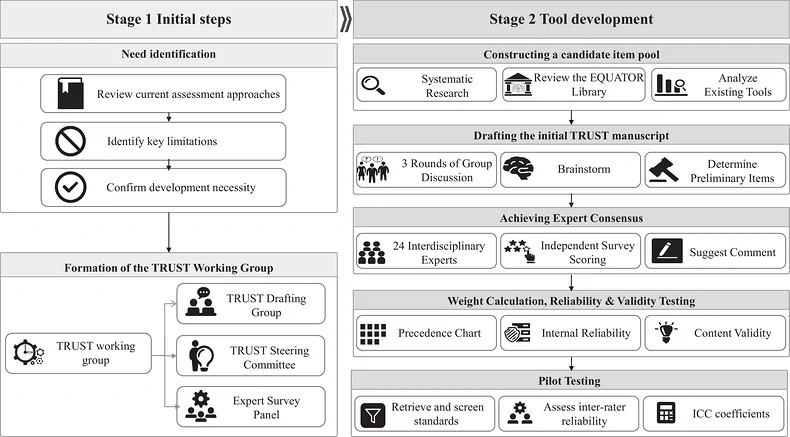
Development process of the TRUST tool. TRUST, the Tool for Rating the qUality of Services Tandards.

We followed the three‐stage framework for developing quality assessment tools proposed by Whiting et al. [22] (Figure 1). Specifically, the framework comprises three stages: Stage 1 “Initial steps,” Stage 2 “Tool development,” and Stage 3 “Dissemination.” Since this study focuses on the foundational development of the TRUST tool, dissemination activities were beyond the scope of the current work. The protocol was registered in the Open Science Framework (osf.io/kx5nv).

### 2.1 Team Assembly

The TRUST working group consisted of three subgroups: the TRUST drafting group, the expert survey panel, and the TRUST steering committee.

The *TRUST drafting group*, composed of the authors of this study, was responsible for conducting the systematic review, and drafting and revising the items of the tool.

The *expert survey panel* comprised 24 experts from diverse disciplines who participated in the structured online expert survey process (Table 1). Experts were recruited through purposive invitation according to their disciplinary background and experience in service standard development, standard evaluation, evidence‐based methodology, healthcare services, social services, or related fields. Several panelists had expertise spanning multiple disciplines; the fields listed in Table 1 represent their primary areas of specialization. A total of 24 experts were invited, all of whom agreed to participate and completed the survey, corresponding to an acceptance rate of 100%. Before participation, all invited experts were asked to disclose any potential conflicts of interest relevant to the development of the TRUST tool. No conflicts of interest were reported.

**TABLE 1:** Characteristics of the expert survey panelists.

Characteristics	Number
Highest education level	
Doctoral degree	17
Master's degree	4
Bachelor's degree	3
Region	
Elsewhere	4
China	20
Professional title	
Senior level (e.g., Full Professor, Department Director)	11
Associate senior level (e.g., Associate Professor)	9
Mid‐level (e.g., Assistant Professor, Research Associate)	4
Field of expertise	
Social services (healthcare management, social work, public health policy)	4
Health science	4
Pedagogy	1
Care service	2
Psychology	2
Methodology	6
Other (ethnology, traditional Chinese medicine, nursing, health management, and medical ethics)	5
Years of work experience	
Less than 5 years	1
5–10 years	4
11–20 years	10
21–30 years	6
More than 30 years	3

While selecting the panelists, we prioritized disciplines such as healthcare services and social services because these areas have comparatively well‐developed traditions of evidence‐based standard development, making them an appropriate starting point for identifying foundational quality dimensions for the preliminary TRUST framework. This choice was aligns with the objective of the present study, which focuses on early conceptual development rather than on establishing full validity across all highly technical or regulatory service sectors.

The *TRUST steering committee* oversaw the overall development process to ensure that the work proceeded efficiently and in an organized manner. Its responsibilities included coordinating participant engagement, organizing the work of the drafting group, arranging the expert survey, and planning the future dissemination and updating of TRUST after its release.

### 2.2 Tool Development

In Stage 2 (tool development), we constructed a pool of candidate items, conducted expert consensus, developed a draft version of the tool, and established the scoring and weighting methods.

#### 2.2.1 Construction of Candidate Item Pool

Following the guidance of Moher et al. [23] for reviewing the literature during reporting tool development, we first undertook a structured review of evidence sources to generate a pool of candidate items. At this stage, the purpose was to identify potentially relevant appraisal domains, items, and conceptual components. Therefore, instead of performing a new bibliographic database search, we used previously published systematic reviews as the primary source of evidence [24, 25]. We searched PubMed, Web of Science, CNKI, and Wanfang Database to identify reviews. In addition, consistent with the recommendation of Moher and colleagues, we reviewed representative reporting guidance documents indexed in the EQUATOR Library [26]. The search cutoff date was September 30, 2024.

Tools and frameworks were considered eligible for item generation if they met the following criteria: (1) they were designed to guide the development, reporting, appraisal, or evaluation of normative documents related to service provision; (2) they contained explicit domains, items, or criteria relevant to the intrinsic quality of such documents; and (3) their full text was accessible for structured extraction. To reduce the risk of missing critical items, two members of the TRUST drafting group independently screened the retrieved records and assessed potentially relevant full texts for eligibility. Disagreements were resolved through discussion.

To generate the candidate items, two investigators separately extracted the following information from the included sources using a standardized approach: the name of the tool, domains, individual items or criteria, and structural characteristics relevant to the evaluation of standards. The results of the independent extraction were then cross‐checked item by item, and discrepancies in wording, interpretation, item boundaries, or domain classification were resolved through discussion within the drafting group until agreement was reached.

#### 2.2.2 Expert Consensus Process

After the preliminary tool items had been formulated, an online expert survey was conducted. Experts were asked to rate the importance of each item on a 5‐point scale ranging from 1 (not important) to 5 (very important), suggest additional items, and provide written comments independently.

Expert authority coefficient (*Cr*) was determined as the arithmetic average of the coefficient of judgment (*Ca*) and the coefficient of familiarity (*Cs*), that is, *Cr* = (*Ca* + *Cs*)/2 [27]. *Ca* represents the evidence when expert makes a judgment, while *Cs* represents the degree of the expert's familiarity with the problem. *Ca* was calculated based on four components: practical experience, theoretical analysis, reference to the literature, and subjective judgment. The degree of influence of each component was classified into three levels (high, medium, and low), which were assigned the following scores: practical experience 0.5, 0.4, 0.3; theoretical analysis 0.3, 0.2, 0.1; reference to domestic and international literature 0.1, 0.1, 0.1; and subjective feeling 0.1, 0.1, 0.1. *Cs* was assessed on a five‐level scale: very familiar (0.9), familiar (0.7), neutral (0.5), unfamiliar (0.3), and very unfamiliar (0.1).

For this phase, sufficient consensus was predefined as meeting all quantitative (mean score ≥ 4.00, full‐score ratio ≥ 0.80, and coefficient of variation ≤ 0.25) and qualitative (no substantial unresolved conceptual disagreement regarding item content, scope, or framework structure in the accompanying free‐text comments) criteria. A second survey round would be undertaken only if one or more items failed to meet the retention criteria, or if the experts’ qualitative comments indicated the need for substantive conceptual revision.

To test the reliability and validity of the tool, the within‐group agreement coefficient (*Rwg*) [28, 29, 30], the Cronbach's *α* coefficient, Item‐level Content Validity Index (I‐CVI), and Scale‐level Content Validity Index/Average method (S‐CVI/Ave) were calculated. I‐CVI is defined as the number of experts considered relevant to the item divided by the total number of experts; and S‐CVI/Ave as the sum of I‐CVI indices across all items divided by the total number of items. When the total number of experts is more than 6, I‐CVI ≥0.78 and S‐CVI/Ave ≥0.90 are considered acceptable [31].

#### 2.2.3 Calculation of Scoring Rules and Weights

The Precedence Chart (PC) method was used to calculate the weight of each item. The PC method is a subjective weighting approach that assigns numerical values through pairwise comparisons. In a precedence chart, the left column and the top row of the chart represent the comparators and the comparands, respectively [15]. In the pairwise comparisons, a value of “1” indicates that the item in the row is more important than that in the column, a value of “0” indicates it is less important, and a value of “0.5” indicates equal importance [32]. The values in each row were summed to obtain the relative total value for each item. The weight of each item was then calculated by dividing its total value by the sum of the total values for all items [33]. For this weighting step, pairwise comparisons were anchored to the mean importance scores of the 17 items obtained from the online expert survey. We did not ask the panelists to explicitly compare all items pairwise, as this would have required an excessive number of comparisons per participant, substantially increasing the workload on the respondents, and potentially reducing the quality and completeness of the responses.

For each pair of items, the item with the higher mean importance score was assigned a value of 1, the lower‐scoring item was assigned 0, and pairs with identical mean scores (rounded to three decimal places) were assigned 0.5. The completed precedence chart was then reviewed by the TRUST drafting group for accuracy and consistency, after which total values and normalized item weights were calculated.

Compared with other weighting methods, the PC method offers several advantages. Unlike the analytic hierarchy process (AHP), which generally requires that the number of indicators being compared pairwise does not exceed nine [34], the PC method is more flexible. Moreover, unlike objective weighting methods such as the entropy method, the PC method avoids the issue of data convergence by allowing weights to be assigned based on expert judgment, and it accounts for the practical value of indicators rather than relying solely on statistical properties. Based on these considerations, this study adopted the more straightforward PC method.

#### 2.2.4 Pilot Testing

Following the development of the preliminary TRUST framework, we conducted a pilot testing study to obtain initial empirical evidence regarding the tool's operability and inter‐rater reliability in real‐world application [19]. The pilot study was undertaken using the *Transparent Registry for Unified Standards and Testing Platform* developed by our team [35].

The pilot sample comprised service standards published between January 1 and December 31, 2025. For Chinese standards, periodic searches were carried out in the Wanfang database according to Chinese disciplinary classifications; and for international standards, potentially eligible documents were identified through regular Google Scholar alerts using “guideline” and “standard” as keywords, as well as through ongoing monitoring of major international guideline platforms, including the National Institute for Health and Care Excellence (NICE) and Guidelines International Network (GIN). The identified documents were regularly screened and uploaded to the platform.

We included all publicly available service standards published in 2025, which fall within domains such as social policy, psychology, education, management, economics, public health, nursing, integrative care, and social work that contained sufficient information for TRUST assessment. Industrial and technical standards were excluded. When duplicate records or multiple versions of the same standard were identified, the latest or most complete one was retained.

Before the two investigators formally commenced scoring, the research team convened a calibration meeting to discuss the scoring rationale, decision boundaries, and principles for resolving disagreements across domains and items. To improve consistency during the pilot testing, a four‐tier scoring framework, based on item‐level scores on a scale from 0 to 10 given by the raters, was established. A score of 0–3 indicated insufficient evidence that the quality dimension was addressed; >3–5 indicated some evidence but with substantial omissions; >5–7 indicated adequate evidence with minor omissions; and >7–10 indicated robust evidence with clear methodological documentation and transparency. Only information explicitly available on the official website, main text, or history records was considered in the assessment.

To evaluate inter‐rater reliability, the two investigators' ratings were compared using the intraclass correlation coefficient (ICC) with 95% confidence intervals (CIs), based on a two‐way random‐effects model for absolute agreement. We calculated both the single‐measure ICC (A,1) for each item and the average‐measure ICC (A,2).

## 3 Results

### 3.1 Constructing a Pool of Candidate Items

Through the search described in the Methods section, we identified 17 relevant tools. After obtaining and reviewing the full text of the documents, we decided to exclude one of them (Ministry of Health and Social Policy of Spain, 2009) [36] for two reasons: (1) it is a methodological manual that describes how to implement clinical practice guidelines, rather than a standardized, operational tool for evaluating implementation quality or outcomes; and (2) its content overlaps strongly with tools already included in our pool. For instance, its framework for analyzing implementation context is explicitly based on the PARIHS model, which was already among our source tools. Finally, we extracted individual items, domains, and structural components from the 16 remaining tools (see Table S1). All extracted content was compiled into a preliminary item pool for TRUST.

Based on the systematic extraction of items, domains, and structural components from the included tools, several consistent themes were identified. The TRUST drafting group found that most instruments emphasized interest‐holder engagement, transparent developmental processes, evidence‐based rigor, external review, applicability, long‐term updating, and social impact.

Between October 2024 and January 2025, the drafting group held three rounds of discussion to conceptually derive and refine these themes into specific evaluation items. The first round focused on mapping extracted themes onto the full life‐cycle logic of service standard development, encompassing formulation, quality assurance, dissemination, updating, and social impact, to establish a theoretically coherent, potentially cross‐sectoral framework rather than merely merging duplicate items across tools. The second round refined wording and eliminated ambiguity; and in the third round, the final set of items was confirmed. The resulting preliminary TRUST tool (see Table S2) contained 17 items organized into five domains: (i) Collaboration, (ii) Scope and Purpose, (iii) Quality Management, (iv) Implementation and Updates, and (v) Social Impact.

### 3.2 Expert Consensus Process

The online expert survey was conducted between January and April 2025. After conducting the survey, we calculated the authority of the experts (*Cr*) as 0.840. Commonly, *Cr* ≥ 0.7 confirms acceptable reliability [37].

Subsequently, the convergence of expert opinions was assessed using the mean score, standard deviation, and coefficient of variation (see Table S3). After the analysis of the results, all items fulfilled the pre‐defined qualitative criteria for consensus, demonstrating strong consensus and alignment among expert opinions. The overall *Rwg* value was 0.994, which demonstrated an exceptionally high level of agreement among the 24 experts. In addition, the qualitative comments mainly addressed wording, clarification of scope, and item consolidation, rather than disagreement about whether some core items should be retained.

Consensus was reached on most items after the expert review. Particularly the importance of multidisciplinary collaboration, interest‐holder engagement, definition of the scope of the standard, consultation processes, dissemination strategies, and update mechanisms had strong agreement among the experts. Based on expert feedback, several modifications, deletions, and consolidations were made.

For *Multidisciplinary Collaboration*, clear multi‐party deliberation rules were added and specific personnel were designated to be responsible for addressing conflicts between evidence‐based experts and service recipients. Regarding *Interest‐Holder Engagement*, the requirement for service recipients to participate in the standard development process was strengthened.

For *scope of the standard*, the indicator “Standard's Domain” was revised to “Specify clearly the target problem or domain for optimization through needs assessment surveys.” Concerning *Public Consultation*, a validation phase was added after drafting, followed by open consultations.

For *dissemination*, the indicator was revised to “Develop diverse promotion strategies for the standard,” mentioning also the use of new media platforms. Finally, the *Update Mechanism* was revised to request the evaluation the need for updates regularly based on the quantity and quality of new evidence, adjust timelines flexibly, and establish a complaint‐handling‐feedback‐update mechanism.

### 3.3 Weight Calculation

The 24 members of the expert survey panel first rated the importance of all 17 items. Subsequently, the TRUST drafting group performed pairwise comparisons of the items based on their mean importance scores to construct the PC weight calculation matrix (see Figure S1). Finally, the values in each row were summed to obtain the total values, from which the weight values for the 17 items were calculated (see Table S4). The top‐weighted items were Registration (Item 6) and Conflicts of Interest (Item 12), each accounting for 11.07% of the total weight.

### 3.4 Reliability and Validity Testing

The Cronbach's *α* coefficient was 0.927 (≥0.7 indicates acceptable consistency). The I‐CVI of the items ranged between 0.96 and 1.00, and the S‐CVI was 0.995, indicating good content validity of the scale [38]. The calculations were based on the draft version of TRUST that was circulated to the expert panel. Although Items 4, 8, 13, and 15 were subsequently refined based on expert comments, these revisions were limited to wording and the conceptual content of the items was not altered.

### 3.5 Description of the TRUST Tool

We ultimately established a comprehensive evaluation system for evidence‐based service standards, covering 17 items. It comprises five domains: Collaboration (28.0% weight), Scope and Purpose (7.6% weight), Quality Management (39.1% weight), Implementation and Updates (15.6% weight), and Social Impact (9.7% weight) (Table 2). The following sections present the details and explanation of each domain and item.

**TABLE 2:** Final content of the TRUST tool.

Domains and items	Weight (%)
Domain 1: Collaboration	
1. Multidisciplinary Collaboration: Involve cross‐domain collaboration, establish clear multi‐party deliberation rules, and designate responsible personnel.	9.34
2. Interest‐Holder Engagement: Consider all interest‐holder groups comprehensively, ensuring full participation of service recipients in the standard development process.	9.34
3. Practical Expertise: Ensure the team contributors possess relevant practical experience and research expertise in the field.	9.34
Domain 2: Scope and Purpose	
4. Definition of Scope: Specify clearly the target problem or domain for optimization through needs assessment surveys.	1.38
5. Necessity Statement: Document the rationale for developing or updating the standard.	6.23
Domain 3: Quality Management	
6. Registration: Register the standard development process on an appropriate platform.	11.07
7. Evidence Basis: The standard must be grounded in high‐quality guidelines or other reliable research evidence.	3.46
8. Evidence Screening: Implement a systematic process for evidence screening and evaluation during development.	6.23
9. Inclusion/Exclusion Criteria: Define explicit criteria for evidence inclusion and exclusion.	3.46
10. Multi‐Interest‐Holder Consensus: Conduct consensus meetings with representatives of multiple interest‐holder groups during the formulation of recommendations.	3.46
11. Public Consultation: Incorporate a validation phase after drafting and conduct open consultations.	0.35
12. Conflicts of Interest: Establish mechanisms to identify, report, and manage conflicts of interest among contributors.	11.07
Domain 4: Implementation and Updates	
13. Dissemination Strategy: Develop diverse promotion strategies for the standard.	6.23
14. Impact Evaluation: Define clear metrics to assess effectiveness and cost‐effectiveness.	7.96
15. Update Mechanism: Evaluate the need for updates regularly based on the quantity and quality of new evidence, adjust the update timelines flexibly, and establish a complaint‐handling‐feedback‐update mechanism.	1.38
Domain 5: Social Impact	
16. Equity and Diversity: Address cultural diversity among service recipients and allow flexible adjustments during implementation.	3.46
17. Social Justice: Explicitly integrate considerations of social equity, justice, and inclusion.	6.23

Collaboration among team members, supported by structured involvement of different interest‐holder representatives, is essential for maintaining high quality of a standard. The items *Multidisciplinary Collaboration, Interest‐Holder Engagement*, and *Practical Expertise* are contained in this domain.

Scope and Purpose focuses on problem definition and the justification of necessity through a needs assessment. This domain contains two items, *Definition of Scope* and *Necessity Statement*.

Quality management focuses on items related to the evidence‐based principle by integrating scientific evidence with professional judgment. Previous research has emphasized that standards have traditionally been based on consensus among experts. There is now growing recognition that standards should be based, when possible, on the systematic identification and synthesis of the best available scientific evidence. This is particularly true for service standards, as relying on consensus among experts has limitations and can lead to flawed conclusions. This domain contains the following items: *Registration, Evidence Basis, Evidence Screening, Inclusion/Exclusion, Multi‐Interest‐Holder Consensus, Public Consultation*, and *Conflicts of Interest*.

Implementation and Updates highlights public consultation processes, diverse dissemination strategies, evaluation metrics, and dynamic update mechanisms. This domain contains the items *Dissemination Strategy, Impact Evaluation*, and *Update Mechanism*.

Social Impact incorporates cultural diversity and social justice into the evaluation process, thereby reflecting the need for cultural relevance and social inclusion. This domain contains the items *Equity and Diversity* and *Social Justice*. Attention should be given to the multidimensional needs of diverse service users. For example, the PROGRESS framework [39] may be adopted. Also, the principle of social justice should be taken into account in the development, so that the standard can contribute to broad social change, promote equity and harmony between diverse groups of users, and thereby fostering a more inclusive social environment.

When using the tool, the raters are requested to assign a score between 0 and 10 for each of the 17 items. The score reflects how well the respective quality dimension is addressed in the standard (0 = not at all; 10 = extremely well). Each score is then multiplied by the item‐specific weight, and the weighted scores are summed up, resulting in an overall score ranging from 0 to 10.

### 3.6 Results of Pilot Testing

To provide an initial empirical assessment of the preliminary TRUST tool, we conducted a pilot application using 51 service standards published in 2025. Of these, 36 (70.6%) were from China and 15 (29.4%) from elsewhere (see Table S5).

The values of ICC (A,1) and ICC (A,2) are presented in Table S6. According to the guidelines proposed by Koo and Li (2016) [39] for single‐measure ICC, values below 0.5 indicate poor reliability, values between 0.5 and 0.75 moderate reliability, values between 0.75 and 0.90 good reliability, and values above 0.90 excellent reliability. In this pilot study, none of the 17 items had an ICC below 0.5. Specifically, 7 items (41.2%) demonstrated good reliability (ICC (A,1) between 0.75 and 0.90), and the remaining 10 items (58.8%) moderate reliability (ICC (A,1) between 0.50 and 0.75). The total scores for the TRUST tool showed good to excellent reliability, with an ICC (A,1) of 0.832 (95% CI: 0.723–0.901) and an ICC (A,2) of 0.908 (95% CI: 0.839–0.948).

## 4 Discussion

This study developed TRUST, a preliminary conceptual and methodological framework targeting the long‐overlooked gap in systematic, evidence‐based quality assessment of service standards. Despite the widespread use of service standards across the public and private sectors, this field that has so far received insufficient academic and practical attention. Grounded in a thorough review of existing assessment frameworks and 16 established evaluation tools, TRUST offers a preliminary structured framework intended for broad cross‐field appraisal that integrates information from multiple assessment domains and lays the groundwork for subsequent psychometric and implementation studies.

A key contribution of this study lies in addressing the fundamental flaws of current service standard assessment practices, which are characterized by their focus on limited aspects and dimensions, over‐reliance on subjective expert opinion, and a lack of tools designed for service standards from multiple fields instead of only specific types like clinical guidelines. Most existing evaluation approaches either focus solely on formal normative compliance or post‐implementation benefits and fail to capture the full scope of service standard quality; the TRUST framework addresses this gap by its conceptually derived five domains that map onto the full life‐cycle logic of service standard development, encompassing formulation, quality assurance, dissemination, updating, and social impact, rather than simply aggregating items from existing tools. This provides a holistic, full‐life‐cycle perspective that moves beyond narrow, single‐dimensional assessment, aligning with the actual development and application logic of service standards.

Compared with sector‐specific tools limited to medicine and healthcare, TRUST is built to be usable in a broad range of fields; hyper‐specialized, domain‐exclusive content has been stripped out. TRUST can thus be deployed across sectors with minimal adaptations, filling the existing demand for universal evaluation benchmarks.

We integrated evidence‐based principles as a core pillar throughout the design of the tool, a feature that is inconsistently applied or entirely absent in most existing service standard assessment frameworks. While prior assessments often prioritize procedural form over the rigor of substantive evidence, TRUST anchors evidence‐based practice as a central component of the evaluation. Additionally, TRUST expands the traditional evaluation boundary to cover the unique public‐oriented attributes of service standards, a dimension rarely emphasized in existing tools, further enhancing its real‐world relevance and practical value.

The study's developmental findings also provide empirical support to these conceptual strengths. The high expert authority coefficient (*Cr* = 0.840), strong within‐group agreement (*Rwg* = 0.994), and high Cronbach's *α* (0.927), I‐CVI, and S‐CVI values demonstrate that TRUST rests on a credible methodological foundation and can reasonably be used in a structured, evidence‐informed manner for appraising service standards. The weighting structure of the final framework offers further insight into the underlying logic of the tool. The prominence of *Quality Management* and *Collaboration* means that the appraisal does not only concern technical content, but also the quality of the development process, the governance structure, and the extent of interest‐holder engagement. This broader orientation aligns with recent calls to strengthen the evidence base of service standards and to give greater weight to implementation, equity, and other public‐facing considerations in evaluative frameworks [19, 20, 25, 40].

Beyond its conceptual contribution, TRUST can also support improvement across multiple stages of the service standard's life cycle. Its uptake in practice, however, will likely depend on how closely its use can be aligned with existing workflows. Many items of TRUST correspond to information that should already be considered in well‐conducted development of standards, such as interest‐holder involvement, evidence justification, implementation planning, and updating arrangements. Therefore, TRUST should not be seen as just an additional administrative task, but rather as a structured guidance to organize and present the methodological information that is often already expected. The main challenge may arise in settings where these processes are less well documented or where methodological support is limited, in which case additional efforts should be made to comprehensively take advantage of the full 17‐item weighted framework.

Hence, a proportionate implementation strategy may be preferable to immediate full‐scale adoption. TRUST could initially be used as a screening and prioritization tool, and comprehensive assessment undertaken only for major revisions, formal endorsement, or cross‐organizational comparison. Feasibility may be further strengthened through a user manual, concise scoring guidance, standardized electronic forms, training of assessors, and integration into existing development and review workflows.

This study has several limitations. First, the present work focused on the initial development of TRUST through evidence synthesis, item generation, expert consultation, and preliminary weighting. Therefore, the main contribution of this study is the establishment of a preliminary conceptual framework and item structure for evaluating service standards, rather than the confirmation of a fully validated assessment instrument. The reported Cronbach's *α* and content validity indices should be interpreted as early‐stage indicators rather than definitive evidence of validity.

Second, because the expert consensus process was conducted as a single‐round online expert survey, panel members did not have the opportunity to reconsider their ratings in light of group feedback. Thus, the observed agreement should be interpreted as preliminary and should be considered alongside the subsequent pilot testing results.

Third, the expert panel and the candidate item pool were weighted toward healthcare and social services, reflecting a practical start concentrated in fields where mature evidence‐based standards are already frequent. While this approach was suitable for identifying foundational quality dimensions, it limits TRUST's applicability to highly technical or regulated sectors. Thus, this study offers a preliminary cross‐sector framework, without guaranteeing universal validity. Future research requires external validation from more experts in fields such as transportation, digital governance, education, and other domains of technical or regulatory services.

Moreover, the resulting weights should be understood as directly anchored to panel ratings, as the weighting procedure was a rule‐based transformation of the experts’ mean importance scores. The practical performance of the weighting requires more empirical testing in future applications.

In conclusion, the present study provides an evidence‐based starting point for TRUST, a comprehensive quality rating tool for service standards. Further work is, however, needed before broader application of TRUST is justified. Future research should apply TRUST to more service standards from diverse sectors and evaluate its reliability, validity, feasibility, and utility in real‐world settings, moving from a conceptual framework to a validated instrument. Additionally, future updates should revisit inaccessible or newly available documents and incorporate external review to further assess the framework's completeness.

## Funding

This study was supported by the Traditional Chinese Medicine Innovation Team and Talent Support Program‐ National Traditional Chinese Medicine Multidisciplinary Cross‐Innovation Team Project (ZYYCXTD‐D‐202401) and National Key R&D Program of China (2025YFC3508700, 2025YFC3508703).

## Conflicts of Interest

All authors declare no conflicts of interest.

## Supporting information




**Supporting File 1**: jebm70173‐supp‐0001‐SuppMat.docx

## Data Availability

Data are available upon reasonable request.
