# Supplementary material for: The Development of the Tool for Rating the qUality of Services sTandards (TRUST)
Source: J Evid Based Med. 2026 Jul 30;19(3):e70173. doi: 10.1111/jebm.70173 (PMC13613253; doi:10.1111/jebm.70173)
Supplement: Supplementary file 1 [file JEBM-19-0-s001.docx]

**Supplementary Materials**

Supplementary Table 1. Analysis and Extraction of Existing Tools^[1]^

| Tool | Aim | Content |
| --- | --- | --- |
| **Appraisal of Methodological Quality** | | |
| Institute of Medicine (IOM)^[2]^ | Proposing eight standards for developing trustworthy guidelines | 8 items: (i) Establishing transparency; (ii) Management of conflict of interest; (iii) Guideline development group composition; (iv) Clinical practice guideline–systematic review intersection; (v) Establishing evidence foundations for and rating strength of recommendations; (vi) Articulation of recommendations; (vii) External review; (viii) Updating |
| Appraisal of Guidelines for Research & Evaluation (AGREE/AGREEII)^[3]^ | Assessing the methodological quality of practice guidelines | 23 items grouped into in 6 domains: (i) Scope and purpose；(ii) Stakeholder involvement；(iii) Rigor of development；(iv) Clarity and presentation；(v) Applicability；(vi) Editorial independence |
| Cluzeau’s “Appraisal Instrument”^[4]^ | Assessing whether developers have minimized the biases inherent in creating guidelines, and addressed the requirements for effective implementation | 37 items describing suggested predictors of guideline quality grouped into three dimensions covering the rigour of development, clarity of presentation (including the context and content) and implementation issues |
| Hayward et al.’s “Structured Abstracts of CPGs”^[5]^ | Proposing recommendations for preparing more informative abstracts of articles describing clinical practice guidelines | 6 items for structured abstracts of CPGs:  (i)Objective; (ii) Options; (iii) Outcomes; (iv) Evidence; (v) Values; (vi) Benefits, harms, and costs |
| Shaneyfelt’s Methodological Standards^[6]^ | A tool to assess methodological quality of the development and reporting of clinical practice guidelines in the peer-reviewed medical literature | 25 items grouped into in 3 domains: (i) Standards of guidelines development and format; (ii) Standards of evidence identification and summary; (iii) Standards on the formulation of recommendations |
| The Reporting Tool for Practice Guidelines in Healthcare (RIGHT)^[7]^ | A reporting guideline for practice guidelines to improve the quality of reporting | 22 items grouped into 7 domains: (i) basic information; (ii) background; (iii) evidence; (iv) recommendations; (v) review and quality assurance; (vi) funding and declaration and management of interests; (vii) other information |
| The Scientific, Transparent, and Applicable Rankings (STAR) system for evaluating the formulation quality of clinical practice guidelines^[8]^ | A comprehensive instrument focusing on the three main dimensions of guideline development: scientificity, transparency, and applicability | 39 items grouped into 11 domains (registration, protocol, funding, working group, conflict of interest, clinical question, evidence, consensus method, recommendation, accessibility and other information) |
| **Appraisal of Implementation Effect** | | |
| Guideline implementation checklist^[9]^ | To support achievement of the National Safety and Quality Health Service (NSQHS) Standards in Australia | Includes 4 dimensions: (i) Governance systems; (ii) Awareness and distribution; (iii) Clinical education; (iv) Quality and safety |
| Guideline implementation evaluation tool^[10]^ | An implementation evaluation tool to promote the overall evaluation of guidelines and to improve their promotion and implementation | 7 items grouped into 5 dimensions: (i) accessibility; (ii) communicability; (iii) performability; (iv) recognizability; (v) applicability |
| Consolidated Framework for Implementation Research (CFIR)^[11]^ | To predict or explain barriers and facilitators to implementation success. | Includes 5 dimensions: (i) innovation, (ii) outer setting, (iii) inner setting,(iv) individuals,(v) implementation process |
| Promoting Action on Research Implementation in Health Systems (PARIHS)^[12]^ | An instrument for the evaluation of clinical practice guidelines | The first 7 items addressed the background characteristics of the respondents.The remaining 16 items were categorized into three domains:(i) use of CPGs (10 questions);(ii) implementation (4 questions);(iii) evaluation of CPGs (2 questions) |
| Goal commitment and implementation of practice guidelines framework^[13]^ | A theoretical framework through which factors facilitating guideline implementation can be explained | Includes 3 dimensions: (i)interactive factors; (ii) external factors; (iii) internal factors; and 2 outcomes:(i) commitment/implementation outcomes; (ii) clinical practice outcome |
| The JBI Implementation Framework ^[14]^ | An overview of the steps required when conducting a small or large-scale evidence implementation project across a health organization. | Includes 7 steps: (i) Identify the practice area;  (ii) Engage change agents;  (iii) Assess context and readiness to change; (iv) Review practice against evidence-based audit criteria;  (v) Implement changes to practice using GRiP;  (vi) Re-assess practice using a follow-up audit;  (vii) Consider sustainability of the project |
| Reach, effectiveness, adoption, implementation and maintenance framework (RE-AIM)^[15]^ | To evaluate public health interventions | Includes 5 dimensions: (i) reach; (ii) efficacy; (iii) adoption; (iv) implementation; and (v) maintenance |
| The Guideline Implementability Framework^[16]^ | An implementability framework of guideline format and content apart from clinical recommendations that are desired by users, or influence use of guidelines | 22 elements grouped into 8 dimensions: (i) adaptability; (ii) usability; (iii) validity; (iv) applicability; (v) communicability; (vi) accommodation; (vii) implementation; (viii) evaluation |
| A guide to the development, implementation and evaluation of clinical practice guidelines^[17]^ | A methodological guide for developing clinical practice guidelines in Australia | Includes 5 chapters: (i) The Guiding Principles;  (ii) Guideline Development;  (iii) Guideline Dissemination and Implementation;  (iv) Guideline Evaluation and Revision;  (v) Legal Considerations |

Supplementary Table 2. Preliminary Development of the Tool Domains and Indicators

| **Domain Indicator** | |
| --- | --- |
| **Collaboration** | |
|  | 1.Multidisciplinary Collaboration: The development of standards should involve collaboration across multiple fields. |
|  | 2.Stakeholder Engagement: The process should comprehensively consider all stakeholders’ interests. |
|  | 3.Practical Expertise: Team members should possess relevant practical and research experience. |
| **Scope & Purpose** | |
|  | 4.Defined Scope: The standard should clearly specify the target problem or domain for optimization. |
|  | 5.Necessity Statement: The rationale for developing or updating the standard should be documented. |
|  | 6.Registration: The standard’s development process should include formal registration. |
| **Quality Management** | |
|  | 7.Evidence Base: Standards should be grounded in high-quality guidelines or reliable research. |
|  | 8.Evidence Screening: A systematic process for evidence screening and evaluation must be implemented. |
|  | 9.Inclusion/Exclusion Criteria: Explicit criteria for evidence inclusion and exclusion. |
|  | 10.Multi-stakeholder Consensus: Whether multi-stakeholder consensus meetings were conducted during the formulation of recommendations. |
|  | 11.Public Consultation: Open consultations should be integrated into the development of standards. |
|  | 12.Conflicts of Interest: Mechanisms to identify, disclose, and manage conflicts of interest among contributors. |
|  | 13.Dissemination Strategy: Concrete strategies for promoting and implementing the standard. |
| **Implementation & Updates** | |
|  | 14.Impact Evaluation: Clear metrics to assess effectiveness and cost-effectiveness. |
|  | 15.Update Mechanism: Plans and mechanisms for regular updates. |
| **Social Impact** | |
|  | 16.Equity & Diversity: Standards should account for cultural diversity among service users and allow flexibility in implementation. |
|  | 17.Social Justice: Standards should explicitly address social equity, justice, and inclusion. |

**Supplementary** **Table 3.** Consensus Convergence

| **Item** | **Mean score** | **Standard Deviation** | **Coefficient of Variation** **(CV)** |
| --- | --- | --- | --- |
| Multidisciplinary Collaboration^(1)^ | 4.875 | 0.338 | 0.069 |
| Interest-holder Engagement^(2)^ | 4.875 | 0.338 | 0.069 |
| Practical Expertise^(3)^ | 4.875 | 0.338 | 0.069 |
| Defined Scope^(4)^ | 4.708 | 0.464 | 0.099 |
| Necessity Statement^(5)^ | 4.792 | 0.415 | 0.087 |
| Registration^(6)^ | 4.917 | 0.282 | 0.057 |
| Evidence Base^(7)^ | 4.750 | 0.442 | 0.093 |
| Evidence Screening^(8)^ | 4.792 | 0.415 | 0.087 |
| Inclusion/Exclusion Criteria^(9)^ | 4.750 | 0.442 | 0.093 |
| Multi-interest-holder Consensus^(10)^ | 4.750 | 0.532 | 0.112 |
| Public Consultation^(11)^ | 4.667 | 0.482 | 0.103 |
| Conflict of Interest^(12)^ | 4.917 | 0.282 | 0.057 |
| Dissemination Strategy^(13)^ | 4.792 | 0.415 | 0.087 |
| Impact Evaluation^(14)^ | 4.833 | 0.381 | 0.079 |
| Update Mechanism^(15)^ | 4.708 | 0.550 | 0.117 |
| Equity & Diversity^(16)^ | 4.750 | 0.442 | 0.093 |
| Social Justice^(17)^ | 4.792 | 0.415 | 0.087 |

**Supplementary Table 4.** The total values from the PC calculation and the resulting weight of each item

| **Item** | **Total Value** | **Weight(%)** |
| --- | --- | --- |
| Multidisciplinary Collaboration^(1)^ | 13.5 | 9.34 |
| Interest-holder Engagement^(2)^ | 13.5 | 9.34 |
| Practical Expertise^(3)^ | 13.5 | 9.34 |
| Defined Scope^(4)^ | 2.0 | 1.38 |
| Necessity Statement^(5)^ | 9.0 | 6.23 |
| Registration^(6)^ | 16.0 | 11.07 |
| Evidence Base^(7)^ | 5.0 | 3.46 |
| Evidence Screening^(8)^ | 9.0 | 6.23 |
| Inclusion/Exclusion Criteria^(9)^ | 5.0 | 3.46 |
| Multi-interest-holder Consensus^(10)^ | 5.0 | 3.46 |
| Public Consultation^(11)^ | 0.5 | 0.35 |
| Conflict of Interest^(12)^ | 16.0 | 11.07 |
| Dissemination Strategy^(13)^ | 9.0 | 6.23 |
| Impact Evaluation^(14)^ | 11.5 | 7.96 |
| Update Mechanism^(15)^ | 2.0 | 1.38 |
| Equity & Diversity^(16)^ | 5.0 | 3.46 |
| Social Justice^(17)^ | 9.0 | 6.23 |

**Supplementary Table 5.** Service standards included in the pilot testing (N = 51)

| Number | Title | Country/Region | Developer Organization/ Source |
| --- | --- | --- | --- |
| 1 | Tobacco: preventing uptake, promoting quitting and treating dependence | UK | NICE |
| 2 | Gambling-related harms: identification, assessment and management | UK | NICE |
| 3 | Overweight and obesity management | UK | NICE |
| 4 | Use of lower-sodium salt substitutes | International | WHO |
| 5 | WHO guideline on contact tracing | International | WHO |
| 6 | Evidence-Based Guidelines for Managing Adolescent Antisocial Behavior | China | Campbell China Network |
| 7 | Competency Training and Certification for Community Education Administrators | China | China Adult Education Association |
| 8 | Service Guidelines for Mental Health Social Work | China | Local government department |
| 9 | Training and Certification for Community Education Teachers | China | China Adult Education Association |
| 10 | Adapting risk assessments to a changing terrorism landscape: Revising the extremism risk guidance | UK | J Forensic Sci. |
| 11 | Adoption: Access to Information and Intermediary Services A Practice Guide | UK | University of East Anglia |
| 12 | ISNS General Guidelines for Neonatal Bloodspot Screening 2025 | Multi-country (including New Zealand, USA, Pakistan) | Int. J. Neonatal Screen. |
| 13 | Headaches in over 12s: diagnosis and management | UK | NICE |
| 14 | Abortion care | UK | NICE |
| 15 | Maternal and child nutrition: nutrition and weight management in pregnancy, and nutrition in children up to 5 years | UK | NICE |
| 16 | Falls: assessment and prevention in older people and in people 50 and over at higher risk | UK | NICE |
| 17 | How to Treat Skin Quality: A Consensus-Based Treatment Algorithm and Expert Guidance | Multi-country (including Germany, UK, Brazil) | Journal of Cosmetic Dermatology |
| 18 | Intrapartum care | the UK | NICE |
| 19 | Somatic symptom and related disorders: Guidance on assessment and management for paediatric health care providers | Canada | Canadian Paediatric Society |
| 20 | Artificial intelligence social experiment—Implementation guidelines | China | Local government department |
| 21 | Artificial intelligence—Risk management capability assessment | China | Local government department |
| 22 | Artificial intelligence—Knowledge graph application platform—Part 2:Performance requirements and testing method | China | Local government department |
| 23 | Artificial intelligence—Service capability maturity assessment | China | Local government department |
| 24 | Diagnostic and Assessment Standards for Compulsory Isolated Drug Rehabilitation | China | Local government department |
| 25 | Community-Based Drug Rehabilitation and Recovery – Part 1: Service Standards for Support, Assistance, and Educational Intervention | China | Local government department |
| 26 | Physical Fitness Assessment Standards for Female Individuals Undergoing Compulsory Isolated Drug Rehabilitation | China | Local government department |
| 27 | Standards for Judicial Administrative Guidance and Support in Community-Based Drug Rehabilitation and Recovery | China | Local government department |
| 28 | Standards for the Establishment of Dedicated Cognitive Impairment Care Units in Elderly Care Institutions | China | Local government department |
| 29 | Community and Home-Based Care Service Standards for Older Adults with Cognitive Impairment | China | Local government department |
| 30 | Mental Health Service Standards for Integrated Medical and Elderly Care Institutions | China | Local government department |
| 31 | Mental Health Service Standards for Children in Distress | China | Local government department |
| 32 | Health Management Technical Standards for Primary and Secondary School Students – Part 1: Mental Health | China | Local government department |
| 33 | Social Work Service Standards for Persons with Disabilities | China | Local government department |
| 34 | Care Service Standards for Persons with Mental Disorders in Residential Care Institutions for Persons with Disabilities | China | Local government department |
| 35 | Operational Standards for Daily Living Care in Residential Care Institutions for Persons with Disabilities | China | Local government department |
| 36 | Action Guidelines for Occupational Health Protection of Key Populations – Part 1: General Principles | China | Local government department |
| 37 | Guidelines for Occupational Health Protection of Workers in the Printing Industry | China | Local government department |
| 38 | Management Standards for Occupational Health Training in Employer Organizations | China | Local government department |
| 39 | Standards for the Establishment and Service Provision of Rehabilitation Institutions for Children with Autism Spectrum Disorder | China | Local government department |
| 40 | Nursing Guideline for Patients with Traumatic Shock | China | Local government department |
| 41 | Course development guide for education about standardization—National quality infrastructure and its application | China | Local government department |
| 42 | Guidelines for Door-to-Door Educational Services for Children and Adolescents with Severe Disabilities at the  Compulsory Education Stage in Child Welfare Institutions | China | Local government department |
| 43 | Standards for the Management of Rehabilitation Treatment  Records in Child Welfare Institutions | China | Local government department |
| 44 | Standards for Speech Therapy and Rehabilitation Services in Child Welfare Institutions | China | Local government department |
| 45 | Physical Rehabilitation Training Standards for  Individuals Undergoing Compulsory Isolated Drug Rehabilitation | China | Local government department |
| 46 | Guidelines for Rehabilitation Training for Children with Functional Disabilities in Child Welfare Institutions | China | Local government department |
| 47 | Service Standards for Community-Based Rehabilitation of Individuals with Mental Disorders | China | Local government department |
| 48 | Service Standards for Traditional Chinese Medicine Nursing for Parkinson's Disease | China | Local government department |
| 49 | Basic Standards for Home-Based Elderly Care Bed Services | China | Local government department |
| 50 | Service Standards for Rehabilitation Training for Older Adults with Dementia in Elderly Care Institutions | China | Local government department |
| 51 | Evidence-Based Standards for Dementia Care Services | China | Campbell China Network |

**Supplementary Table 6.** Intraclass correlation coefficients (ICCs) for each item and the total score in the pilot test.

| **Item** | **Two-way Random Effects for Absolute Agreement** | **ICC coefficient** | **95% CI** |
| --- | --- | --- | --- |
| Multidisciplinary Collaboration^(1)^ | ICC(A,1) | 0.714 | 0.548 ~ 0.826 |
|  | ICC(A,K) | 0.833 | 0.708 ~ 0.905 |
| Interest-holder Engagement^(2)^ | ICC(A,1) | 0.502 | 0.265 ~ 0.682 |
|  | ICC(A,K) | 0.668 | 0.419 ~ 0.811 |
| Practical Expertise^(3)^ | ICC(A,1) | 0.841 | 0.737 ~ 0.906 |
|  | ICC(A,K) | 0.913 | 0.848 ~ 0.951 |
| Defined Scope^(4)^ | ICC(A,1) | 0.514 | 0.281 ~ 0.691 |
|  | ICC(A,K) | 0.679 | 0.438 ~ 0.817 |
| Necessity Statement^(5)^ | ICC(A,1) | 0.762 | 0.617 ~ 0.857 |
|  | ICC(A,K) | 0.865 | 0.763 ~ 0.923 |
| Registration^(6)^ | ICC(A,1) | 0.860 | 0.767 ~ 0.918 |
|  | ICC(A,K) | 0.925 | 0.868 ~ 0.957 |
| Evidence Base^(7)^ | ICC(A,1) | 0.707 | 0.537 ~ 0.821 |
|  | ICC(A,K) | 0.828 | 0.699 ~ 0.902 |
| Evidence Screening^(8)^ | ICC(A,1) | 0.804 | 0.679 ~ 0.883 |
|  | ICC(A,K) | 0.891 | 0.809 ~ 0.938 |
| Inclusion/Exclusion Criteria^(9)^ | ICC(A,1) | 0.693 | 0.516 ~ 0.812 |
|  | ICC(A,K) | 0.818 | 0.681 ~ 0.897 |
| Multi-interest-holder Consensus^(10)^ | ICC(A,1) | 0.690 | 0.513 ~ 0.811 |
|  | ICC(A,K) | 0.817 | 0.678 ~ 0.895 |
| Public Consultation^(11)^ | ICC(A,1) | 0.656 | 0.466 ~ 0.788 |
|  | ICC(A,K) | 0.792 | 0.636 ~ 0.881 |
| Conflict of Interest^(12)^ | ICC(A,1) | 0.792 | 0.662 ~ 0.876 |
|  | ICC(A,K) | 0.884 | 0.797 ~ 0.934 |
| Dissemination Strategy^(13)^ | ICC(A,1) | 0.759 | 0.613 ~ 0.855 |
|  | ICC(A,K) | 0.863 | 0.760 ~ 0.922 |
| Impact Evaluation^(14)^ | ICC(A,1) | 0.505 | 0.269 ~ 0.684 |
|  | ICC(A,K) | 0.671 | 0.424 ~ 0.812 |
| Update Mechanism^(15)^ | ICC(A,1) | 0.816 | 0.699 ~ 0.891 |
|  | ICC(A,K) | 0.899 | 0.823 ~ 0.942 |
| Equity & Diversity^(16)^ | ICC(A,1) | 0.553 | 0.330 ~ 0.718 |
|  | ICC(A,K) | 0.712 | 0.496 ~ 0.836 |
| Social Justice^(17)^ | ICC(A,1) | 0.557 | 0.335 ~ 0.721 |
|  | ICC(A,K) | 0.716 | 0.502 ~ 0.838 |
| Total | ICC(A,1) | 0.832 | 0.723 ~ 0.901 |
|  | ICC(A,K) | 0.908 | 0.839 ~ 0.948 |
| *Note:* ICC(A,1) and ICC(A,K) are intraclass correlation coefficients for absolute agreement. ICC(A,1) is the reliability of a single rater; ICC(A,K) is the reliability of the average of K raters (here, K = 2). Values <0.5, 0.5–0.75, 0.75–0.90, and >0.90 indicate poor, moderate, good, and excellent reliability, respectively. | | | |

Supplementary Figure 1. Precedence Chart Weight Calculation Table

| Item | **1** | **2** | **3** | **4** | **5** | **6** | **7** | **8** | **9** | **10** | **11** | **12** | **13** | **14** | **15** | **16** | **17** |
| --- | --- | --- | --- | --- | --- | --- | --- | --- | --- | --- | --- | --- | --- | --- | --- | --- | --- |
| **1** | *0.5* | 0.5 | 0.5 | 1 | 1 | 0 | 1 | 1 | 1 | 1 | 1 | 0 | 1 | 1 | 1 | 1 | 1 |
| **2** | 0.5 | *0.5* | 0.5 | 1 | 1 | 0 | 1 | 1 | 1 | 1 | 1 | 0 | 1 | 1 | 1 | 1 | 1 |
| **3** | 0.5 | 0.5 | *0.5* | 1 | 1 | 0 | 1 | 1 | 1 | 1 | 1 | 0 | 1 | 1 | 1 | 1 | 1 |
| **4** | 0 | 0 | 0 | *0.5* | 0 | 0 | 0 | 0 | 0 | 0 | 1 | 0 | 0 | 0 | 0.5 | 0 | 0 |
| **5** | 0 | 0 | 0 | 1 | *0.5* | 0 | 1 | 0.5 | 1 | 1 | 1 | 0 | 0.5 | 0 | 1 | 1 | 0.5 |
| **6** | 1 | 1 | 1 | 1 | 1 | *0.5* | 1 | 1 | 1 | 1 | 1 | 0.5 | 1 | 1 | 1 | 1 | 1 |
| **7** | 0 | 0 | 0 | 1 | 0 | 0 | *0.5* | 0 | 0.5 | 0.5 | 1 | 0 | 0 | 0 | 1 | 0.5 | 0 |
| **8** | 0 | 0 | 0 | 1 | 0.5 | 0 | 1 | *0.5* | 1 | 1 | 1 | 0 | 0.5 | 0 | 1 | 1 | 0.5 |
| **9** | 0 | 0 | 0 | 1 | 0 | 0 | 0.5 | 0 | *0.5* | 0.5 | 1 | 0 | 0 | 0 | 1 | 0.5 | 0 |
| **10** | 0 | 0 | 0 | 1 | 0 | 0 | 0.5 | 0 | 0.5 | *0.5* | 1 | 0 | 0 | 0 | 1 | 0.5 | 0 |
| **11** | 0 | 0 | 0 | 0 | 0 | 0 | 0 | 0 | 0 | 0 | *0.5* | 0 | 0 | 0 | 0 | 0 | 0 |
| **12** | 1 | 1 | 1 | 1 | 1 | 0.5 | 1 | 1 | 1 | 1 | 1 | *0.5* | 1 | 1 | 1 | 1 | 1 |
| **13** | 0 | 0 | 0 | 1 | 0.5 | 0 | 1 | 0.5 | 1 | 1 | 1 | 0 | *0.5* | 0 | 1 | 1 | 0.5 |
| **14** | 0 | 0 | 0 | 1 | 1 | 0 | 1 | 1 | 1 | 1 | 1 | 0 | 1 | *0.5* | 1 | 1 | 1 |
| **15** | 0 | 0 | 0 | 0.5 | 0 | 0 | 0 | 0 | 0 | 0 | 1 | 0 | 0 | 0 | *0.5* | 0 | 0 |
| **16** | 0 | 0 | 0 | 1 | 0 | 0 | 0.5 | 0 | 0.5 | 0.5 | 1 | 0 | 0 | 0 | 1 | *0.5* | 0 |
| **17** | 0 | 0 | 0 | 1 | 0.5 | 0 | 1 | 0.5 | 1 | 1 | 1 | 0 | 0.5 | 0 | 1 | 1 | *0.5* |

*Note*: This figure presents the pairwise comparisons and weight calculation using the Precedence Chart (PC) method. The left column and the top row represent the comparators and comparands, respectively. The cell values indicate: 1 = the row item is more important than the column item; 0 = the row item is less important; 0.5 = equal importance. The total relative value (TTL) for each item is the sum of its row values. The weight of each item is calculated as its TTL divided by the sum of all TTLs. The figure was constructed by the research group based on the mean importance scores of 17 items rated by 24 experts. (1.Multidisciplinary Collaboration; 2.Interest-holder Engagement; 3.Practical Expertise; 4.Definition of Scope; 5.Necessity Statement; 6.Registration; 7.Evidence Basis; 8.Evidence Screening; 9.Inclusion/Exclusion Criteria; 10.Multi-interest-holder Consensus; 11.Public Consultation; 12.Conflict of Interest; 13.Dissemination Strategy; 14.Impact Evaluation; 15.Update Mechanism; 16.Equity & Diversity; 17.Social Justice.)

1. This table was compiled based on IOM (2011) and Freitas de Mello et al. (2024).
2. **References**

   IOM (Institute of Medicine). Clinical Practice Guidelines We Can Trust. https://nap.nationalacademies.org/resource/13058/Clinical-Practice-Guidelines-2011-Report-Brief.pdf (accessed 5 March 2026)
3. AGREE Collaboration, “Development and Validation of an International Appraisal Instrument for Assessing the Quality of Clinical Practice Guidelines: The AGREE Project,” *Quality & Safety in Health Care* 12, no. 1 (2003): 18–23.
4. F. A. Cluzeau, P. Littlejohns, J. M. Grimshaw, G. Feder, and S. E. Moran, “Development and Application of a Generic Methodology to Assess the Quality of Clinical Guidelines,” *International Journal of Quality Health Care* 11, no. 1 (1999): 21–28.
5. R. S. Hayward, M. C. Wilson, S. R. Tunis, E. B. Bass, H. R. Rubin, and R. B. Haynes, “More Informative Abstracts of Articles Describing Clinical Practice Guidelines,” *Annals of Internal Medicine* 118, no. 9 (1993): 731–737.
6. T. M. Shaneyfelt, M. F. Mayo-Smith, and J. Rothwangl, “Are Guidelines Following Guidelines? The Methodological Quality of Clinical Practice Guidelines in the Peer-Reviewed Medical Literature,” *JAMA* 281, no. 20 (1999): 1900–1905.
7. Y. Chen, K. Yang, A. Marušic, et al., “A Reporting Tool for Practice Guidelines in Health Care: The RIGHT Statement,” *Annals of Internal Medicine* 166, no. 2 (2017): 128–132.
8. N. Yang, W. Zhao, Y. Pan, et al., “Development of a Ranking Tool for the Scientificity, Transparency, and Applicability of Clinical Practice Guidelines(STAR),” *Natl Med J China* 102, no. 30 (2022): 9.
9. Queensland Health. Guideline implementation checklist. https://www.health.qld.gov.au/__data/assets/pdf_file/0014/154022/o-implementationchecklist.pdf (accessed 5 March 2026)
10. Y. Jin, Z. Zhao, C. Huang, et al., “Development and validation for evaluation of an evaluation tool for guideline implementation,” *Chinese Journal of Evidence-Based Medicine* 22, no. 1 (2022): 111–119.
11. Consolidated Framework for Implementation Research. https://cfirguide.org/ (accessed 5 March 2026)
12. C. Bahtsevani, A. Willman, A. Khalaf, and M. Östman, “Developing an Instrument for Evaluating Implementation of Clinical Practice Guidelines: A Test-Retest Study,” *Journal of Evaluation in Clinical Practice* 14, (2008): 839–846.
13. A. F. Chou, T. E. Vaughn, K. D. McCoy, and B. N. Doebbeling, “Implementation of evidence-based practices: Applying a goal commitment framework,” *Health Care Management Review* 36, no. 1 (2011): 4-17.
14. JBI. Section 2: Evidence implementation projects using an evidence-based audit and feedback approach: the JBI Implementation Framework. https://jbi-global-wiki.refined.site/space/JHEI (accessed 6 May 2026)
15. R. E. Glasgow, T. M. Vogt, and S. M. Boles, “Evaluating the public health impact of health promotion interventions: the RE-AIM framework,” *American Journal of Public Health* 89, no. 9 (1999): 1322-1327.
16. A. R. Gagliardi, M. C. Brouwers, V. A. Palda, et al., “How can we improve guideline use? A conceptual framework of implementability,” *Implementation Science* 6, no. 26 (2011).
17. National Health and Medical Research Council (NHMRC). A guide to the development, implementation and evaluation of clinical practice guidelines. https://www.nhmrc.gov.au/sites/default/files/images/a-guide-to-the-development-and-evaluation-of-clinical-practice-guidelines.pdf (accessed 8 March 2026)
